# Speech perception and hearing effort using a new active middle ear implant audio processor

**DOI:** 10.1007/s00405-021-07207-4

**Published:** 2021-12-07

**Authors:** Torsten Rahne, Laura Fröhlich, Luise Wagner, Miriam Hannah Kropp, Alexander Müller

**Affiliations:** 1 Department of Otorhinolaryngology, Head and Neck Surgery, University Hospital Halle (Saale), Halle (Saale), Germany; 2 ORL Department Friedrichshain Clinic, Vivantes Hearing Center, Berlin, Germany

**Keywords:** Speech perception, Listening effort, Vibrant Soundbridge, SAMBA 2, Outcome, Questionnaires

## Abstract

**Purpose:**

The Vibrant Soundbridge (VSB) was introduced in 1996, and the fourth generation of the audio processor recently released. This clinical study evaluates the audiological performance and subjective satisfaction of the new SAMBA 2 audio processor compared to its predecessor, SAMBA.

**Method:**

Fifteen VSB users tested both audio processors for approximately 3 weeks. Air conduction and bone conduction thresholds and unaided and aided sound field thresholds were measured with both devices. Speech performance in quiet (Freiburg monosyllables) and noise (OLSA) was evaluated as well as subjective listening effort (ACALES) and questionnaire outcomes (SSQ12 and APSQ). In addition, data from 16 subjects with normal hearing were gathered on sound field tests and ACALES.

**Results:**

Both audio processors showed substantial improvement compared to the unaided condition. The SAMBA and SAMBA 2 had comparable performance in sound filed thresholds, while the SAMBA 2 was significantly better in speech in quiet, speech in noise, reduced listening effort, and improved subjective satisfaction compared with the SAMBA.

**Conclusion:**

The SAMBA 2 audio processor, compared to its predecessor SAMBA, offers improved performance throughout the parameters investigated in this study. Patients with a VSB implant would benefit from an upgrade to SAMBA 2.

**Supplementary Information:**

The online version contains supplementary material available at 10.1007/s00405-021-07207-4.

## Introduction

The Vibrant Soundbridge (VSB, Med-El, Innsbruck, Austria) is a semi-implantable active hearing device [1, 2] indicated for patients with sensorineural-, mixed-, or conductive hearing loss who cannot use conventional hearing aids due to medical reasons or insufficient benefit [3]. The VSB consists of the implantable Vibrating Ossicular Prosthesis (VORP) and the externally worn audio processor (AP). Sound is received through the AP´s two microphones, then subjected to signal processing and transcutaneously transmitted over the coil section to the VORP implant. The AP is held onto the intact skin via magnetic attraction. The VSB was introduced in 1996 and proved to be a safe and effective device for hearing loss treatment [4, 5].

The first VSB audio processor (AP404) was equipped with omnidirectional microphones only. Research showed that ominidirectional microphones are beneficial for spatial hearing and listening to music while directional microphones improve performance in listening situations in noisy environments [6, 7]. The second-generation “Amadé” was released in 2009 and enabled users to manually switch between omnidirectional and directional settings. With improved signal processing algorithms and an intelligent system that automatically switches between the omni- and directional setting, the third-generation “SAMBA” AP, released in 2015, was more convenient for the user and provided better results in challenging noise situations compared to the previous AP generation [8]. The latest generation, the “SAMBA 2” AP was introduced in 2020. A chip provides two additional frequency and compression bands, and improved signal processing features. The SAMBA 2 AP is intended to automatically detect the listening environment, adapt the directional microphones, focus on desired speech, detect interfering speech, and reduce background noise [9].

As hearing device technology develops, benefit is becoming possible in increasingly complex listening situations. Research has tried to continuously create and adapt tests to recreate real-life scenarios in controlled settings. The Oldenburg Sentence test (OLSA, HoerTech, Oldenburg, Germany) incorporates a range of different noise signals that can be used to create a complex listening situation. In this study, we used the OLSA combined with multiple noise sources to test hearing performance in complex setups [3, 10].

Poor speech signal quality due to disturbing background noise or hearing impairment requires the patient to invest more cognitive resources and is perceived to be more effort [11]. Thus, listening effort is another relevant parameter to evaluate the success of the treatment and can be accessed objectively by dual-task paradigm [12] and pupillometry [13] or subjectively using the Adaptive Categorical Listening Effort Scaling test (ACALES, HörTech, Oldenburg, Germany). The ACALES test was previously used to evaluate hearing aid performance [14, 15]. Speech in noise is presented using an adaptive procedure to control the signal-to-noise ratio while the listener rates the subjectively perceived listening effort [16].

The aim of this study was to compare the speech perception in noise in two complex setups, subjective listening effort, and subjective satisfaction between the previous and latest generation of the VSB audio processor. The secondary objective was to test subjects with normal hearing on the OLSA- and ACALES-setups, to enable a comparison between treatment results and normal hearing.

## Material and methods

### Procedure

This study was conducted as a bicentric prospective study with a single subject, repeated measures design. It was approved by the Ethics Committee of the Medical Faculty of the XXX (EK number: 2020-121). Written informed consent regarding study participation and data protection was obtained from all patients before enrolment into the study. Performance of the SAMBA 2 audio processor was compared to the SAMBA audio processor, using audiometric tests and questionnaires at the initial visit (unaided condition), and 3 and 6 weeks after the initial visit, with minor deviations due to local COVID restrictions. A SAMBA and SAMBA 2 were fitted to each subject in a randomized order for consecutive usage during the time period investigated.

Base line audiological assessment consisted of unaided air conduction (AC), bone conduction (BC) and uncomfortable loudness (UCL) threshold measurements. At all three appointments, warble tone (WT) thresholds, speech recognition in quiet (Freiburger Monosyllables) and in noise (OLSA), and hearing effort in noise (ACALES) were measured. Self-reported outcome and satisfaction was assessed by the Audio Processor Satisfaction Questionnaire (APSQ) and the Spatial and Qualities of Hearing Scale Questionnaire (SSQ12) for both aided conditions (SAMBA and SAMBA 2).

### Subjects

Thirty two subjects were enrolled in this study and divided into 2 groups, with 16 subjects in the normal hearing (NH) group and 16 in the Vibrant Soundbridge (VSB) group. The subjects implanted with VSB used either a VORP 502 or VORP 503 implant and the Amadé or SAMBA audio processor for at least 6 months. The NH group was tested to generate reference data for the OLSA and the ACALES test setups. The study was conducted between November 2020 and July 2021. 16 VSB users were initially enrolled in the study; one subject chose to terminate the study prematurely after the SAMBA 2 device trial, reporting that the device was too loud. The patient declined refitting and was not interested in continuing to participate in the study. The mean age of the 15 subjects with hearing loss (5 female, 10 male) was 64 ± 12 years (range: 37–82 years) at the time of consent.

The mean age in the NH group (14 female, 2 male) was 32.0 ± 4.8 years (range: 24–42 years) at the time of consent and 4 were tested at the right ear and 12 at the left ear. See Table 1.

**Table 1:** Subject characteristics

Subject no	Age (years)	Sex	Implant side	Coupler	Own audio processor	Test AP	PTA4 AC ipsilateral dB HL	PTA4 BC ipsilateral dB HL	PTA4 AC contralateral dB HL	PTA4 BC ipsilateral dB HL	PTA4 UCL ipsilateral dB HL
P1	53	Female	Right	Symphonics	SAMBA Lo	SAMBA Lo/SAMBA 2 Lo	49	29	46	34	> 110
P2	55	Male	Right	CliP-Coupler	SAMBA Hi	SAMBA Hi/SAMBA 2 Hi	98	40	19	19	> 110
P3	75	Male	Right	CliP-Coupler	SAMBA Hi	SAMBA Hi/SAMBA 2 Hi	63	31	70	> 38	> 110
P4	69	Male	Left	Direct (w/o)	SAMBA Hi	SAMBA Hi/SAMBA 2 Hi	78	49	28	29	> 110
P5	37	Male	Left	RW-Soft-Coupler	SAMBA Hi	SAMBA Hi/SAMBA 2 Hi	99	36	11	11	> 110
P6	53	Female	Left	RW-Soft-Coupler	SAMBA Hi	SAMBA Hi/SAMBA 2 Hi	79	51	> 91	> 61	> 110
P7	61	Male	Left	CliP-Coupler	SAMBA Lo	SAMBA Lo/SAMBA 2 Lo	70	33	78	43	99
P8	73	Male	Left	CliP-Coupler	SAMBA Hi	SAMBA Hi/SAMBA 2 Hi	56	35	50	30	93
P9	69	Female	Left	Direct (w/o coupler)	SAMBA Hi	SAMBA Hi/SAMBA 2 Hi	76	44	21	19	> 110
P10	72	Female	Left	RW-Coupler	SAMBA Hi	SAMBA Hi/SAMBA 2 Hi	> 78	43	34	25	> 110
P11	62	Male	Left	OW-Coupler	SAMBA Hi	SAMBA Hi/SAMBA 2 Hi	90	40	30	23	> 110
P12	54	Male	Left	CliP-Coupler	SAMBA Hi	SAMBA Hi/SAMBA 2 Hi	64	39	26	20	> 110
P13	68	Male	Left	OW-Coupler	SAMBA Lo	SAMBA Lo/SAMBA 2 Lo	64	26	45	33	> 110
P14	82	Female	Right	RW-Coupler	SAMBA Hi	SAMBA Hi/SAMBA 2 Hi	71	43	55	38	> 110
P15	79	Male	Right	CliP-Coupler	SAMBA Hi	SAMBA Hi/SAMBA 2 Hi	76	45	> 81	> 50	> 110
Mean	64.1						73.9	38.8	45.7	31.3	
SD	12.1						14.1	7.2	24.9	13.3	

Patients (P) air conduction (AC), bone conduction (BC) and uncomfortable loudness (UCL) thresholds. If a hearing threshold was out of the audiometers limit, the result was replaced by the limit level in dB HL for data analysis (see red marked values in Table S2). For calculation of PTA4, if replaced values were used for calculation, the results were indicated with a “greater-than sign” (“>”). If the patients UCL threshold was beyond the audiometers limit, the limit level of 110 dB HL was used for audio processor fitting. PTA4 is the, average of 0.5, 1, 2 and 4 kHz

### Fitting of the audio processors

Both the SAMBA and SAMBA 2 audio processors were fitted precisely to the patient, following a first fitting session using DSL I/O for SAMBA and DSL version 5 for SAMBA 2. This fitting was based on the Vibrant Soundbridge Vibrogram and uncomfortable loudness (UCL) thresholds with 80–100% acclimatization. If the subject’s UCL threshold was out of the audiometer’s limit (110 dB HL) and thus not measurable, 120 dB HL was used as the UCL threshold for fitting. Two SAMBA and SAMBA 2 audio processor models, Lo and Hi, were used in the study with different maximum gains. The software programs applied for the SAMBA audio processor were Connexx (Sivantos, Erlangen, Germany) version 6.5.5 and SYMFIT (Med-El, Innsbruck, Austria) version 7.0 or 7.0.1. The SAMBA 2 audio processor was fitted with SYMFIT, version 8.0.

### Audiometric methods

Pure-tone audiometry was performed at 0.25, 0.5, 1, 1.5, 2, 3, 4, 6 and 8 kHz. Uncomfortable loudness (UCL) was evaluated using pure tones via headphone on the ipsilateral ear.

Sound field thresholds were tested using warble tones (WT) from a loudspeaker 1 m in front of the subject (S_0_) at 0.25, 0.5, 1, 1.5, 2, 3, 4, 6 and 8 kHz. Word recognition score in quiet (WRS) was measured using German Freiburg monosyllables at 65 dB SPL from the front (S_0_). The speech perception threshold in noise, in which the subject correctly understands 50% of the words in a presented sentence (SRT_50_), was measured with the German Oldenburg Sentence test (OLSA) in two setups [17], OLSA_Olnoise_ and OLSA_ISTS_. To simulate a complex listening situation with speech shaped stationary noise and interfering speech coming from the back, the speech signal was presented from the front (S_0_) in an adaptive procedure. Simultaneously, continuous background noise was played from three loudspeakers 1 m away from the subject’s head, positioned at 120°, 180° and 240°, with the volume held constant at 65 dB SPL. In the OLSA_Olnoise_ setup, Olnoise (N), speech-shaped noise derived from a male voice, was used as background noise (S0°, N120°, N180°, N240°). In setup OLSA_ISTS_, the International Speech Test Signal (ISTS) (Holube et al. 2010), a single female talker of interfering non-stationary speech noise that consists of sentence fragments from six languages (American English, Arabic, Chinese, French, German, and Spanish), was presented from 180°. Olnoise was presented from 120° to 240° (S0°, N120°, ISTS180°, N240°).

The subjects rated their subjective listening effort via the ACALES [14]. The speech signal was presented from the front loudspeaker (S0°), and the ISTS single-talker interferer was presented from the back (ISTS180°). The speech level and thus the signal-to-noise ratio (SNR) was changed adaptively with the ISTS fixed at 65 dB SPL and presented continuously. After presentation of two OLSA sentences in noise, participants were asked to rate their listening effort on a scale of 8 answers ranging from “no effort” to “only noise”. Each round of two sentences was presented at a different signal-to-noise ratio. The software calculated a listening effort function; the cutting point at 4 effort scale categorical units (ESCU) represented the SNR at which moderate effort was applied. This SNR-cut at 4 ESCU was used for data evaluation. All sound field measurements were performed monaurally; within the VSB group the contralateral ear was occluded with ear plugs (110, 3 M, Berkshire, United Kingdom) when necessary. Subjects with normal hearing (AC thresholds  ≤ 20 dB HL at 0.5, 1, 1.5, 2, 3 and 4 kHz) were evaluated in the unaided condition; the evaluation took place at one visit, with one ear occluded for comparability. This data were used as a reference. Occlusion was done using ear plugs.

### Subjective satisfaction

Hearing-related satisfaction was determined using the Audio Processor Satisfaction Questionnaire (APSQ) and the Speech, Spatial and Qualities of Hearing Scale Questionnaire (SSQ12). The APSQ consists of 15 items divided into 3 dimensions (comfort, social life, and usability) and has a minimum and maximum range between zero and ten [18]. In 2013, Noble et al. validated the short version of the Speech, Spatial and Qualities of Hearing Scale Questionnaire (SSQ), named SSQ12, using a scale from 0 to 10 [19]. The SSQ12 consists of 12 items and is subdivided into 3 dimensions (speech, spatial and qualities).

### Statistical analysis

The Kolmogorov–Smirnov Test and the Shapiro–Wilk Test were used to test for normal distribution. One-way ANOVA with Tukey’s multiple comparison test was used to compare the results in the unaided, SAMBA-aided and SAMBA 2-aided conditions. For questionnaire outcomes, two-way ANOVA with Sidak’s multiple comparison test was used to compare subscore (dimension) results between the SAMBA and SAMBA 2. The level of statistical significance was set to *p*  < 0.05. Descriptive statistics, such as mean, standard deviation (SD) and/or median with range (minimum and maximum), and 25–75th percentiles were used to present the results. For statistical analysis and graphs, GraphPad Prism 7 (GraphPad Software, Inc., San Diego, California, United States) was used. If the hearing threshold was out of the audiometers limit, the result was recorded as out of limit (ool) and replaced by the limit level in dB HL for data analysis (see red marked values in Table S2). For calculation of averages like PTA4, if replaced values were used for calculation, those were indicated with a “greater-than sign” (“>”), see Table 1. For OLSA results equal to or higher than 5 dB SNR the recommended procedure according to the minimal reporting standard by Maier et al. [20] was applied. In these cases, a best-case estimate of 5 dB SNR was used for data analysis.

## Results

### Pure tone audiometry

The mean PTA4 (threshold average at 0.5, 1, 2, 4 kHz) air conduction (AC) threshold was 74 ± 14 dB HL. The bone conduction (BC) threshold was 38.8 ± 7.2 dB HL. The AC threshold of the contralateral ear was 46 ± 26 dB HL and the BC threshold was 29 ± 11 dB HL. The UCL threshold of the ipsilateral ear was out of the audiometer limit in at least one PTA4 frequency for 13 of 15 subjects. In the NH group, the mean PTA4 AC threshold was 4.8 ± 2.2 dB HL in the ipsilateral (ear to be occluded for further tests) and 4.0 ± 3.2 dB HL in the contralateral ear. Occluding the ipsilateral ear with an ear plug resulted in a mean PTA4 AC threshold of 36.5 ± 6.1 dB HL. Thus, the occlusion effect was on average 31.7 ± 6.0 dB. For individual results, see supplementary material Table S1.

### Sound field thresholds

**Fig. 1:**
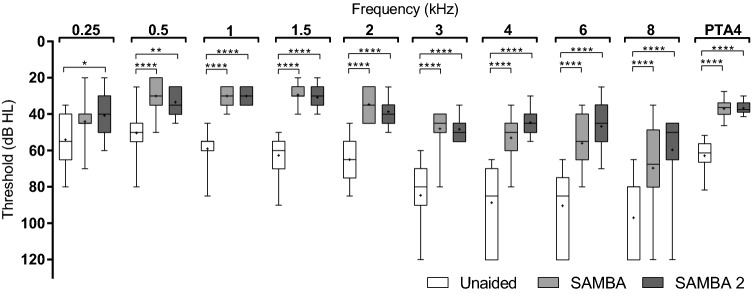
Sound field thresholds of subjects with hearing loss with the SAMBA and SAMBA 2 audio processors and in the unaided condition

Sound field thresholds improved significantly with both devices, from an unaided PTA4 of 66 ± 10 dB SPL to aided PTA4 thresholds of 36.9 ± 5.0 dB SPL with SAMBA (*p*  < 0.0001) and 36.7 ± 3.4 dB SPL with SAMBA 2 (*p*  < 0.0001), one-way ANOVA (F (1.7, 23.1)  =  96.1; *p*  < 0.0001). No significant difference in sound field thresholds was found between the devices (*p*  = 0.9835). Figure 1 shows the individual results per frequency.

Warble tones were presented from the front (S0°). Box plot: whiskers represent minimum and maximum; “+” symbol represents the mean; box: 25–75th percentiles; horizontal line: median. Two-way ANOVA (F (9, 419)  =  43; *p*  < 0.0001).

### Vibrogram

**Fig. 2:**
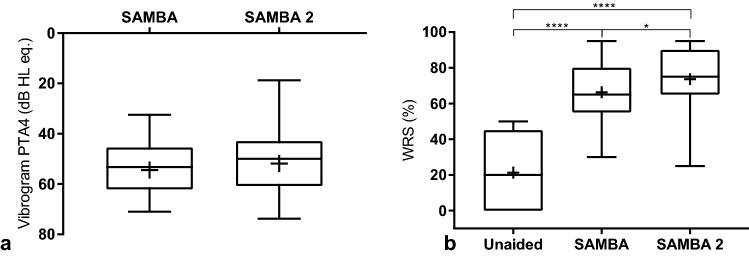
**a** Vibrogram results, **b** speech in quiet with Freiburg monosyllables at 65 dB SPL (S0°). Box plot: whiskers represent minimum and maximum; “+” symbol represents the mean; box: 25–75th percentiles; horizontal line: median

The vibrogram (VIB) threshold was 54 ± 10 for SAMBA and 52 ± 14 for SAMBA 2 (Fig. 2a). The difference was not statistically significant (Wilcoxon test, *p*  = 0.0533).

### Speech audiometry

The mean word recognition score (WRS) in quiet was 21 ± 20% in the unaided condition. The WRS improved significantly with both devices and reached 66 ± 20% with SAMBA (*p*  < 0.0001) and 74 ± 19% using SAMBA 2 (*p*  < 0.0001). Thus, the SAMBA 2 speech perception in quiet results were 7.3% higher than those with the SAMBA, and the difference was statistically significant (*p*  = 0.0390), one-way ANOVA (F (1.3, 18.7)  =  68.9; *p*  < 0.0001). See Fig. 2b.

**Fig. 3:**
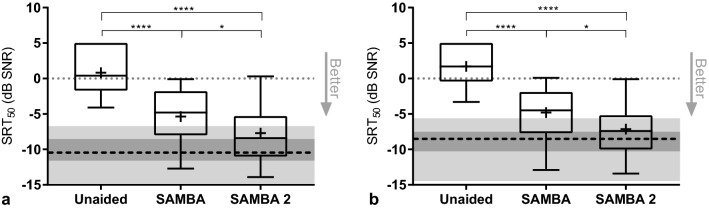
**a** OLSA_Olnoise_, Olnoise (N), speech-shaped noise, was used as background noise (S0°, N120°, N180°, N240°). **b** OLSA_ISTS_, the International Speech Test Signal (ISTS), a single talker interfering speech noise, was presented from 180°. Olnoise was presented from 120° to 240° (S0°, N120°, ISTS180°, N240°). Box plot: whiskers: minimum and maximum; “+” symbol represents the mean; box: 25–75th percentiles; horizontal line: median. The light gray horizontal bar represents the minimum and maximum, the dark gray bar represents the 25–75th percentiles around the median (dashed line) of the normal hearing results

The SRT50 in noise using the OLSA_Olnoise_ setup (S0°, N120°, N180°, N240°) was 0.8 ± 3.1 dB SNR in the unaided condition, − 5.4 ± 3.7 dB SNR with SAMBA and − 7.7 ± 3.9 dB SNR with SAMBA 2. The difference compared with the unaided threshold was significant for both the SAMBA (*p*  = 0.0001) and SAMBA 2 (*p*  < 0.0001), see Fig. 3a. The difference of 2.4 dB between the mean SRT50 thresholds of the devices was statistically significant (*p*  = 0.0264), one-way ANOVA (F (1.7, 24.1)  =  38.4; *p*  < 0.0001). At the individual level, 8 of 15 subjects improved with SAMBA 2 by more than 2 dB (range: 2.3–8.5 dB improvement) compared to SAMBA. Two subjects performed better (> 2 dB improvement) with SAMBA compared to using SAMBA 2 (2.1 dB and 3.6 dB improvement).

The normal-hearing group scored a mean SRT50 in noise in the OLSA_Olnoise_ setup of − 10.6 ± 2.3 dB SNR in the unaided condition with one ear occluded. Out of the hearing-impaired group, eight subjects reached thresholds within the one-time standard deviation (1 ×  SD) range of the normal hearing reference (≥ − 8.3 dB SNR) using SAMBA 2 and 3 subjects using SAMBA. Nine subjects reached thresholds within the one and a half standard deviation (1.5 ×  SD) range of the normal hearing reference (≥ − 7.2 dB SNR) using SAMBA 2 and 4 subjects with SAMBA. 87% of subjects improved with SAMBA 2 compared to SAMBA and 62% of them improved by more than 2 dB (range: 2.3–8.5 dB).

Using the OLSA_ISTS_ setup (S0°, N120°, ISTS180°, N240°), the SRT50 in noise was 1.7 ± 2.6 dB SNR unaided, aided with SAMBA − 4.8 ± 3.7 dB SNR and − 7.1 ± 3.7 dB SNR aided with SAMBA 2. The improvement over the unaided condition was significant for both SAMBA (*p*  < 0.0001) and SAMBA 2 (*p*  < 0.0001), see Fig. 3b. The 2.3-dB difference between the mean results of both devices was significant (*p*  = 0.0427), one-way ANOVA (F (1.8, 25.7)  =  41.4; *p*  < 0.0001). However, at the individual level, 8 of 15 subjects improved with SAMBA 2 by more than 2 dB (range: 2.3–10.0 dB improvement) compared with SAMBA. Two subjects performed better (> 2 dB improvement) with SAMBA compared to SAMBA 2 (3.0 dB and 3.3 dB improvement).

The normal-hearing group scored a mean SRT50 in noise in the OLSA_ISTS_ setup of − 9.1 ± 2.3 dB SNR in the unaided condition with one ear occluded. Out of the hearing-impaired group, eight subjects reached thresholds within the 1xSD range of the normal hearing reference (≥− 6.8 dB SNR) using SAMBA 2 and 3 using SAMBA. Eleven subjects reached thresholds within the 1.5 ×  SD range of the normal hearing reference (≥ − 5.7 dB SNR) using SAMBA 2 and 4 subjects reached this mark with SAMBA. 87% of subjects improved with SAMBA 2 compared to SAMBA and 62% of them improved by more than 2 dB (range: 2.3–10.0 dB).

### Subjective listening effort

The result of the ACALES subjective listening effort test was a SNRcut at 4 ESCU of 10.8 ± 5.7 dB SNR in the unaided condition, 4.6 ± 5.1 dB SNR using SAMBA and 1.3 ± 4.7 dB SNR with the SAMBA 2. Meaning, on average the subjects felt moderate effort to understand the sentences, unaided when the speech was by 10.8 dB louder than the noise. With SAMBA the signal-to-noise ratio at which moderate effort was used was 4.6 dB SNR and with SAMBA 2 the SNR decreased to 1.3 dB.

**Fig. 4:**
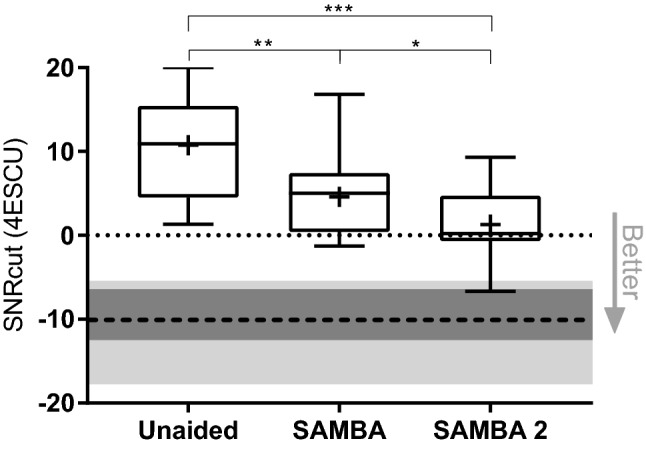
Adaptive categorical listening effort scaling (ACALES). ACALES was performed with speech (OLSA sentences), presented from the front (S0°) at various levels and interfering speech (ISTS) from the back (ISTS180°) at 65 dB SPL continuously. SNRcut at 4ESCU represents the cutting point of the listening effort function at the point of moderate difficulty in dB SNR. Box plot: whiskers: minimum and maximum; “+” symbol represents the mean; box: 25–75th percentiles; horizontal line: median. The light gray horizontal bar represents the minimum and maximum, the dark gray bar represents the 25–75th percentiles around the median (dashed line) of the normal hearing results

The difference over the unaided condition was significant with both SAMBA (*p*  = 0.0039) and SAMBA 2 (*p*  = 0.0004), see Fig. 4. The subjective listening effort improved significantly by 3.3 dB SNR with SAMBA 2 compared to the result with SAMBA (*p*  = 0.0366), one-way ANOVA (F (1.6, 22.7)  =  19.9; *p*  < 0.0001). 80% of subjects improved with SAMBA 2 compared to SAMBA and 83% of them improved by more than 2 dB (range: 2.6–8.7 dB).

Normal-hearing subjects scored a mean SNRcut at 4 ESCU of − 9.9 ± 3.7 dB SNR in the unaided condition with one ear occluded.

### Subjective satisfaction

**Fig. 5:**
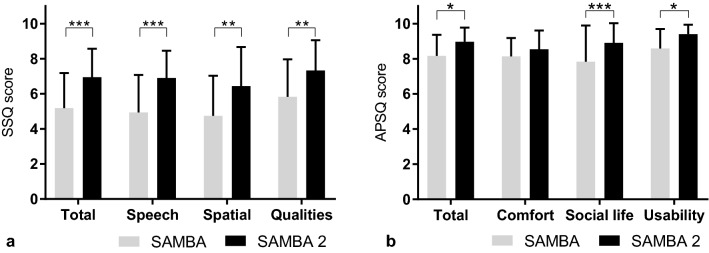
**a** Speech, spatial and qualities of hearing scale (SSQ12), **b** audio processor satisfaction questionnaire (APSQ). Bars and whiskers represent the mean and standard deviation

The SSQ hearing-specific questionnaire scores were: mean total score 5.2 ± 2.0 points, speech subscore 4.9 ± 1.1 points, spatial subscore 4.7 ± 2.3 points and qualities subscore 5.8 ± 2.1 points using SAMBA. With SAMBA 2 the mean total score was 7.0 ± 1.6 points, 6.9 ± 1.6 points in the speech subscore, 6.4 ± 2.2 points in the spatial subscore and 7.3 ± 1.7 points in the qualities subscore. With SAMBA 2 the speech (*p*  = 0.0027), spatial (*p*  = 0.0017) and qualities (*p*  = 0.0063) subscores as well as the total score (*p*  = 0.0010) improved significantly compared to using SAMBA, two-way ANOVA (F (3, 56)  = 0.80; *p*  = 0.4469). See Fig. 5a.

The scores of the APSQ audio processor-specific questionnaire were: mean total score 8.2 ± 1.2 points, comfort dimension 8.1 ± 1.0 points, social life dimension 7.8 ± 2.1 points and usability dimension 8.6 ± 1.1 points using SAMBA. With SAMBA 2 the scores were: mean total score 9.0 ± 0.8 points, 8.5 ± 1.1 points in the comfort dimension, 8.9 ± 1.1 points in the social life dimension and 9.4 ± 0.5 points in the usability dimension. The social life (*p*  = 0.0005) and usability (*p*  = 0.0116) dimensions and the total score (*p*  = 0.0134) improved with SAMBA 2 significantly compared to SAMBA, two-way ANOVA [F (3, 56)  =  1.2; *p*  =  0.3280]. See Fig. 5b.

## Discussion

We compared the audiological performance and subjective satisfaction of the SAMBA 2 audio processor with its predecessor SAMBA. Both audio processors showed substantial improvement compared to the unaided condition. Comparing SAMBA and SAMBA 2 directly, our results showed comparable performance in sound field thresholds between the devices and better results with SAMBA 2 in speech tests in quiet and noise. As the maximum output and gain are similar for both devices [9], comparable results were expected. Word recognition score in quiet was on average 7.3% higher with SAMBA 2 compared with SAMBA and speech understanding in noise improved on average by more than 2 dB SNR using the new generation audio processor compared with SAMBA. We speculate that the new chip technology and improved signal processing of the SAMBA 2 AP are the main reasons for the elevated audiological performance. The speech in noise setup (OLSA_ISTS_), which uses single talker interfering speech (ISTS) from the back, was incorporated as the most challenging speech test scenario within this study. When comparing both setups (OLSA_Olnoise_ vs OLSA_ISTS_) in the unaided condition, the mean difference was 0.6 dB SNR within the patient group and 1.5 dB SNR within the NH group. Higher thresholds (more difficulty) were measured in the OLSA_ISTS_ setup. Subjects using SAMBA 2 were more often within the 1.5 ×  SD range of the normal hearing reference data, when tested with the more complex OLSA_ISTS_ setup compared to the OLSA_Olnoise_ setup. While using SAMBA, only a few subjects had thresholds within this range. Thus, users of SAMBA 2 seem to benefit from the new signal processing features in general compared to SAMBA, especially in more challenging conditions. However, the WRS was below 60% for 3 out of 15 participants using SAMBA and for 1 participant using SAMBA 2. According to the AWMF guideline [21] for cochlear implant (CI) treatment, those patients would be within the audiological indication for a CI implantation. Since speech perception outcome strongly depends on the mechanical coupling of the actuator to the ossicles or the round window [22–24] and the mean difference between the VIB and BC threshold was  > 20 dB in three patients with SAMBA and in three patients using SAMBA 2, optimizing the coupling could be an option to potentially further reduce the speech perception gap. The coupling efficiency, however, does not explain the difference between the two audio processors. Here, the influence of different sound qualities or a difference in handling could potentially have influenced the respective speech perception outcome, which would also be reflected by the larger range of WRS and SRT50 results.

The subjective listening effort, tested by ACALES, improved significantly using SAMBA 2 (*p*  = 0.0366) compared to performance with the predecessor device SAMBA. Although with advanced technology hearing impaired people are achieving hearing thresholds closer to normal, the gap we see in our data regarding listening effort is substantial. On average subjects with normal hearing felt moderate effort was needed for understanding sentences at − 10 dB SNR. Unaided subjects with hearing impairment felt moderate effort on average at  + 11 dB SNR. With SAMBA this was reduced to 4.6 dB SNR and with SAMBA 2 down to 1.3 dB SNR. While subjects with hearing impairment might hear reasonably well using a hearing system, more effort and concentration is required over the day compared to people with normal hearing [25, 26]. Assessment of listening effort in addition to conventional audiometry and a complex noise setup for speech perception testing gave a more comprehensive evaluation of our patient’s needs. As more sophisticated and better technology becomes available in hearing devices, we believe the test setup should be designed adequately to avoid possible saturation effects, to show differences and to detect treatment gaps. In our study, patients with SAMBA 2 show near normal aided speech in noise results while room for improvement regarding listening effort remained. We hope the manufacturer will target this topic in the following audio processor development.

Subjective satisfaction numerically improved with SAMBA 2 in all domains of both the audio processor specific and the hearing related questionnaire and was higher compared to SAMBA. The statistically significant improvement in all domains of the SSQ12 questionnaire correlates with the improved results in the audiological speech tests. To our knowledge, the normal-hearing reference data generated in the speech in noise and ACALES setups mentioned above were the first to be published.

A limitation we see in this study is that the performance of the new SAMBA 2 AP was not tested in all indications of this device. SAMBA 2 can be used by Vibrant Soundbridge users as well as with the Bonebridge implant. The benefit with SAMBA 2 in Bonebridge users with conductive hearing loss and/or single-sided deafness remains unstudied. A trail time longer than 3 weeks would be beneficial. Normal-hearing data were generated monaurally by plugging the subject`s contralateral ear with an ear plug, thus contribution of the contralateral ear could not be totally eliminated.

## Conclusion

The Vibrant Soundbridge patient population in this study fitted with the SAMBA 2 audio processor showed clinically relevant improvements in speech in quiet and speech in noise performance as well as reduced listening effort compared to the SAMBA. Subjective satisfaction was significantly elevated in all except for one domain using SAMBA 2 compared to SAMBA. With SAMBA 2, many patients can achieve a better approximation to the hearing ability of healthy people. The results of this study conclude that many VSB implant users would benefit from the SAMBA 2 audio processor.

## Supplementary Information

Below is the link to the electronic supplementary material.

Supplementary file 1 (XLSX 30 KB)

## Data Availability

For data transparency, the raw data (Tables S1, S2) are provided in the supplementary material.
